# Microarray dataset of transient and permanent DNA methylation changes in HeLa cells undergoing inorganic arsenic-mediated epithelial-to-mesenchymal transition

**DOI:** 10.1016/j.dib.2017.05.002

**Published:** 2017-05-10

**Authors:** Meredith Eckstein, Matthew Rea, Yvonne N. Fondufe-Mittendorf

**Affiliations:** University of Kentucky Department of Molecular and Cellular Biochemistry, United States

**Keywords:** Inorganic arsenic, DNA methylation, Infinium Methylation EPIC Bead-Chip, Epithelial-to-mesenchymal transition, Epigenetics

## Abstract

The novel dataset presented here represents the results of the changing pattern of DNA methylation profiles in HeLa cells exposed to chronic low dose (0.5 µM) sodium arsenite, resulting in epithelial-to-mesenchymal transition, as well as DNA methylation patterns in cells where inorganic arsenic has been removed. Inorganic arsenic is a known carcinogen, though not mutagenic. Several mechanisms have been proposed as to how inorganic arsenic drives carcinogenesis such as regulation of the cell׳s redox potential and/or epigenetics. In fact, there are gene specific studies and limited genome-wide studies that have implicated epigenetic factors such as DNA methylation in inorganic arsenic-mediated epithelial-to-mesenchymal transition (EMT). However, genome-wide studies about the impact of 1) chronic, low-dose inorganic arsenic exposure on DNA methylation patterns during inorganic arsenic-induced epithelial-to-mesenchymal transition, and 2) the removal inorganic arsenic (reversal) on DNA methylation patterns, is lacking. For this dataset, two replicates were performed with each of the samples – non-treated, inorganic arsenic-treated, and reverse-treated cells. We provide normalized and processed data, and log2 fold change in DNA methylation. The raw microarray data are available through NCBI GEO, accession number GSE95232 and a related research paper has been accepted for published in Toxicology and Applied Pharmacology (Eckstein et al., 2017) [1].

## Specifications Table

**Table:** 

Subject area	*Biology*
More specific subject area	*Epigenetics and Toxicology*
Type of data	Text, raw data files
How data was acquired	Infinium Methylation EPIC Bead Chip Methylation Array
Data format	*Raw, filtered*
Experimental factors	*HeLa cells were exposed to 0.5 µM sodium arsenite, mimicking the levels of arsenic in the water supply of Eastern Kentucky (in particular). Cells that had undergone the epithelial-to-mesenchymal transition from inorganic arsenic exposure then had the sodium arsenite removed and continued to be grown to see if the removal of the environmental toxicant could reverse some of the methylation and gene expression changes observed. Non-treated HeLa cells were used as a control to which DNA methylation changes could be compared.*
Experimental features	*We analyzed DNA methylation changes genome-wide by profiling HeLa cells that were exposed to 0.5 µM sodium arsenite, as well as cells that were treated and had arsenic removed using the methylation array.*
Data accessibility	*Microarray data has been deposited into the NCBI GEO database (Accession number*GSE95232*)*

## **Value of the data**

• Genome-wide assessment of DNA methylation changes in inorganic arsenic-mediated epithelia-to-mesenchymal transition cells and reversal of inorganic arsenic-treated cells.
• DNA methylation profiling in cells in response to chronic, low dose inorganic arsenic exposure.
• Implication of DNA methylation as an epigenetic contributor to inorganic-arsenic mediated epithelial-to-mesenchymal transition.
• Valuable for understanding genome-wide epigenetic implications of chronic, low dose exposure to inorganic arsenic, a common environmental toxicant.

## 1 Data

The Infinium Methylation EPIC BeadChip Array profile data are provided as MINiML and TXT files deposited into NCBI GEO, accession number GSE95232. Genomic DNA was extracted from non-treated, inorganic arsenic-treated, and inorganic arsenic reverse-treated HeLa cells, bisulfite converted, and applied to the BeadChip Array. Two replicates of each sample were performed. The BeadChip Array detected DNA methylation status genome wide of non-treated, inorganic arsenic-treated, and inorganic arsenic reverse-treated HeLa cells. Gene ontology analysis of the differentially methylated genes revealed that many of the genes had oncogenic signatures according to the Gene Set Enrichment Analysis (Supplemental Tables 1–3). Specifically, many of them are connected to KRAS signaling in several types of cancer in epithelial cells such as those of the lung, breast, and kidney tissues. A more complete description of this data can be found in Eckstein et al. (2017) [1].

## 2 Experimental design, materials and methods

### 2.1 HeLa cell growth conditions and treatment

HeLa cells were grown as previously described in [3], [4]. Reverse-treated cells were treated with 0.5 µM sodium arsenite for 36 days, at which point they were treated with water instead of sodium arsenite (reverse-reated cells). These reverse-treated cells were harvested 10 days after reversal of treatment.

### 2.2 Infinium MethylationEPIC Bead Chip – methylation array analysis

*Sample preparation*. Genomic DNA from non-treated, treated, and reverse treated cells was extracted using the Qiagen DNeasy Blood and Tissue kit. DNA was initially bisulfite converted using the Zymo EZ-96DNA Methylation Kit (Catalog #D5004) Deep-Well Format. DNA was treated with sodium bisulfilte causing unmethlylated cytosines to convert to uracil while keeping methylated cytosines unchanged. Then 4ul (equivalent to 750 ng DNA) of the bisulfite converted DNA was used as input for the Illumina Infinium HD Methylation Array. During this process, the bisulfite converted DNA samples were denatured, neutralized, and prepared for amplification. After amplification, the amplified DNA was enzymatically fragmented and precipitated. The resuspended DNA samples were then dispensed onto Illumina׳s Infinium MethylationEPIC BeadChip, where they underwent a series of washing, extension, and staining procedures. The BeadChips are then coated for protection and scanned on the Illumina HiScanHQ. Once scanning is completed, the data is uploaded into GenomeStudio for preliminary analysis and QC.

### 2.3 Data analyses

For statistical analysis, beta values were calculated. The methylation levels of CpGs were described as beta values (0–1) representing the calculated level of methylation (0–100%). We had two technical and two biological replicates processed by chip technique. The Pearson correlation coefficients (PCCs) were >0.99 for all the replicates, confirming a good level of reproducibility for the chip process and indicating that the observed differential methylation between the cells (treatments) represented true biological differences. Functional normalization was performed using home scripts ‘Minfi preprocessFunNorm’ which does the background normalization. Additionally, the dye correction was performed using noob. All CpG sites with detection value>0.05, CpG sites with SNPs, as well as probes predicted to hybridize to more than one genomic location [2] were removed.
