# Supplementary material for: Microarray dataset of transient and permanent DNA methylation changes in HeLa cells undergoing inorganic arsenic-mediated epithelial-to-mesenchymal transition
Source: Data Brief. 2017 May 10;13:6–9. doi: 10.1016/j.dib.2017.05.002 (PMC5443927; doi:10.1016/j.dib.2017.05.002)
Supplement: Supplementary file 1 — Supplementary material [file mmc1.docx]

**Conflict of Interest Statement.** The authors state no conflicts of interest pertaining to this work.
