# Supplementary material for: Microarray dataset of transient and permanent DNA methylation changes in HeLa cells undergoing inorganic arsenic-mediated epithelial-to-mesenchymal transition
Source: Data Brief. 2017 May 10;13:6–9. doi: 10.1016/j.dib.2017.05.002 (PMC5443927; doi:10.1016/j.dib.2017.05.002)
Supplement: Supplementary file 2 — Supplementary material [file mmc2.docx]

***Table 1.*** *List of differentially methylated genes in iAsT cells that have an oncogenic signature (910 genes)*

A4GALT,ABAT,ABCA8,ABCC8,ABHD5,ABLIM2,ACAT2,ACHE,ACOX3,ACP6,ACSM3,ACVR2A,ADAM10,ADAM19,ADAM22,ADAMTS8,ADAMTS9,ADAP1,ADCY10,ADCYAP1R1,ADD2,AFF3,AGA,AGL,AGPAT4,AHCY,AHR,AIM1,AJAP1,AK4,AKAP9,AKT3,ALB,ALDH1A2,ALLC,ALOX12B,ALPL,AMD1,ANK1,ANK2,ANK3,ANKRD11,ANKRD32,ANKS1B,ANO1,ANO2,ANXA13,AOAH,APOA1,APOE,ARAP3,ARC,ARG2,ARHGAP12,ARHGAP22,ARHGAP24,ARHGAP8,ARHGEF10L,ARHGEF12,ARHGEF3,ARL4A,ARMC2,ARMCX1,ARPP21,ARSD,ASAP2,ASPA,ASRGL1,ASTN2,ATF1,ATG13,ATP1A3,ATP1B4,ATP4A,ATP5A1,ATP7B,ATP8A1,ATP8A2,ATP8B2,ATR,ATXN7,ATXN8OS,AUTS2,AXIN1,B3GNT2,BAG2,BAG6,BAMBI,BAZ2B,BBS12,BCAS1,BCL11B,BCL2,BEAN1,BEND5,BHLHE22,BMI1,BMP3,BMP6,BMP7,BPI,BRF1,BTBD11,BTG3,BZW1,C11orf83,C14orf132,C17orf79,C18orf1,C1orf21,C3,C6orf103,C6orf64,CACNA1C,CACNA1H,CACNB3,CADM1,CADPS2,CALB2,CALD1,CALN1,CAMK2A,CAND2,CARD14,CARS2,CASC1,CATSPERB,CBLN1,CCDC85B,CCDC88C,CCNT2,CCR6,CD14,CD24,CD248,CD44,CD48,CD8A,CDC14A,CDC25B,CDC7,CDCA3,CDH11,CDH12,CDH13,CDH22,CDH4,CDK5,CEACAM3,CELF2,CENPE,CENPO,CEP41,CHD9,CHRDL1,CHRM1,CHRNA2,CHRNA7,CHST10,CHST11,CHST12,CHST8,CIITA,CLCA4,CLCNKB,CLDN17,CLN6,CLSTN2,CMKLR1,CMPK2,CNO,CNTNAP2,COCH,COL18A1,COL1A1,COL6A2,COX6B2,CPPED1,CPT1A,CRABP2,CREB1,CRELD1,CREM,CRIP2,CRMP1,CSF2RB,CTBP2,CTDSPL,CTF1,CTNNA2,CTNNA3,CTNND2,CUL4A,CWC22,CX3CR1,CXCL13,CXCR2,CYLD,CYP11B2,CYP1B1,CYP24A1,CYP2B6,CYP4B1,CYP7B1,CYR61,CYTIP,DAAM2,DAZL,DCC,DDAH1,DDX3Y,DDX60,DGKB,DIMT1,DIP2C,DKK2,DLEC1,DLG2,DLGAP2,DMRT1,DNAH8,DNAJA1,DNAJB11,DNAJC12,DOCK2,DOT1L,DPAGT1,DPF3,DPP6,DPYS,DPYSL3,DSCAM,DSP,DTX4,DUOX1,DUOX2,DUSP10,E2F5,EBF1,EFHD1,EGFR,EGR1,EHBP1,EIF4E,ELAVL4,ELK3,ELL2,ELMO1,ELN,EMR2,EMR3,ENC1,ENPP5,EOMES,EPB41,EPHA4,EPHA5,EPHB2,EPHB3,EPHX2,ERCC6L,ERLIN2,ESR1,ESRP1,ESRRG,EXOSC7,EYA2,EYA3,F11,FABP7,FAM167B,FAM189A1,FAM3C,FAM49A,FAM76A,FANCA,FARP1,FAT3,FBLN5,FBN1,FBXO17,FBXO42,FCHSD1,FEV,FEZ1,FGA,FGF12,FGF3,FHL2,FLI1,FLJ13197,FMO4,FMO6P,FOXM1,FOXN3,FRAS1,FRMD4A,FRZB,FSHB,FSHR,FSTL4,FXYD2,FXYD6,FZD6,G6PD,GABBR2,GABRA5,GABRB1,GABRB3,GAL,GALC,GALNT5,GALNT6,GALNT7,GALNTL4,GAPVD1,GATA3,GATA4,GATC,GBP4,GCDH,GCLC,GDAP1L1,GFPT2,GHR,GINS1,GINS2,GLB1L,GNA14,GNG7,GOLGA4,GPC4,GPR144,GPR3,GPR31,GPR37,GPR63,GPRC5B,GPX5,GRAMD3,GREM1,GRIA1,GRID1,GRIN2A,GRK4,GRM8,GRN,GRPR,GSPT2,GTF2H5,HAS1,HDGFRP3,HEPH,HIST1H3H,HJURP,HLADOA,HLCS,HMGA2,HMGN2,HMOX1,HMP19,HOXB3,HOXC11,HOXD8,HPN,HRH1,HS3ST1,HS3ST2,HSD17B2,HSD3B7,HSPA12A,HSPA1B,HTATIP2,HTR1E,HTR4,HTR6,HVCN1,HYDIN,IFI6,IGSF9B,IKZF2,IL36A,IL7R,ILF2,IMPA1,INSM2,INSR,INTS9,IPO4,IQGAP2,IRAK1BP1,IRX5,ISG20L2,ISL1,ITGA5,ITGA9,ITGAE,ITIH2,KALRN,KAT5,KAZN,KCNA10,KCNA2,KCNB2,KCNC2,KCNH2,KCNIP1,KCNIP4,KCNJ3,KCNK10,KCNMA1,KCNN1,KCNQ1,KCNV1,KDELR3,KIAA0125,KIAA0513,KIAA1009,KIAA1456,KIRREL3,KLF3,KLHL1,KLHL21,KLHL28,KLK10,KLK6,KLK8,KTI12,LAMA2,LAMA3,LAMA4,LAMC3,LAMP3,LAPTM4A,LARGE,LCMT1,LFNG,LHX6,LILRA4,LIMCH1,LIN7C,LMBR1,LMCD1,LMO2,LMO7,LMOD1,LOC157627,LOR,LOXL1,LOXL2,LPAR3,LPIN1,LPIN3,LRP2,LSAMP,LYPD1,LYRM5,MAB21L1,MAGI2,MAML3,MAP3K1,MAPK10,MAPK8IP3,MAPT,MARCH3,MAST4,MBD4,MBTPS2,MCF2L,MCM5,MECOM,MED17,MEGF6,METTL20,MFI2,MFNG,MGA,MGLL,MICB,MIIP,MINPP1,MITF,MOBP,MPPED1,MPPED2,MRTO4,MS4A4A,MS4A6A,MSMB,MSMO1,MSR1,MTHFD2,MTPAP,MTSS1,MTTP,MTUS2,MYCN,MYH14,MYH15,MYH3,MYL2,MYO10,MYO1D,MYOM2,MYT1L,NAGA,NBEA,NCK2,NCOA7,NDRG2,NDUFV1,NEB,NECAP1,NEIL1,NEIL3,NELL1,NELL2,NES,NEU3,NEURL,NEUROG3,NFATC2,NFIL3,NIPAL3,NKX21,NLRP1,NLRP2,NOL4,NOS1,NPHS1,NPTXR,NPY2R,NR3C1,NRG1,NRL,NRN1,NRP2,NRXN1,NRXN3,NT5DC2,NTF3,NTM,NTN1,NTNG2,NUP155,NVL,ODZ2,ODZ4,OLFM1,OPCML,OPRM1,OSBP2,OTX2,PACRG,PAH,PAIP2B,PAK3,PANX1,PAPD4,PAPOLA,PAQR5,PARK2,PARM1,PARP11,PARP12,PAX1,PAX3,PAX4,PCBP3,PCCB,PCDH17,PCDH9,PCDHA2,PCDHA9,PCP4,PDE11A,PDE1B,PDE4C,PDE4D,PDE4DIP,PDGFRL,PDHA2,PDLIM3,PDLIM5,PDPN,PDYN,PDZRN3,PGAP1,PHACTR3,PHB,PHF14,PHF16,PHF20,PHIP,PHLDA1,PHOSPHO1,PIEZO1,PIGV,PIH1D1,PIK3CD,PIK3CG,PIN4,PINK1,PKHD1,PKIB,PLA2G2A,PLA2G4C,PLCE1,PLCH1,PLEKHA1,PLEKHO2,PLIN3,PLLP,PLP1,PLS3,PLSCR2,PMFBP1,PMP2,PMS1,PNOC,PPCS,PPFIA2,PPFIA3,PPIL4,PPP1R9A,PPP3CA,PPP4R4,PPP5C,PRAC,PRAMEF12,PRDM1,PRKCQ,PRMT10,PRMT8,PROL1,PSD,PSD4,PSMD13,PTEN,PTK7,PTMS,PTN,PTPN12,PTPRD,PTPRE,PTPRG,PTPRM,PTPRN2,PTPRR,PTPRU,PTPRZ1,PTS,PVRL3,PYHIN1,PYY,RAB17,RAB31,RAD18,RAD51AP1,RAD52,RAD54B,RAD54L,RALGDS,RAPGEF5,RASA3,RASGEF1A,RASSF1,RASSF4,RBFOX1,RBMS1,RBP3,RBP7,RCAN2,RCHY1,RELN,RET,RFX4,RGS6,RHBDD3,RIBC2,RIMS1,RIMS2,RND3,RNF11,RNF141,RNF144A,RNF220,ROPN1B,RPIA,RPP25,RPS6KA2,RPS6KB2,RTDR1,RTN1,RUNX1,RXRA,RYR2,RYR3,SACS,SALL3,SAMD11,SARDH,SARM1,SATB1,SATB2,SCAF11,SCAP,SCGN,SCN1B,SCN2A,SCN8A,SCN9A,SCNN1B,SCUBE1,SDK2,SEMA3E,SEMA3F,SEPT7,SERAC1,SERHL2,SERPINA12,SERPINA3,SERPING1,SERPINI2,SESN1,SEZ6L,SGCG,SGMS1,SHANK1,SHC2,SHH,SHISA2,SHOX2,SHROOM2,SIM2,SIX1,SIX4,SKAP1,SLC12A5,SLC13A3,SLC16A3,SLC17A6,SLC19A3,SLC22A17,SLC22A6,SLC24A3,SLC24A5,SLC26A10,SLC2A9,SLC30A1,SLC30A4,SLC35C2,SLC35F4,SLC4A4,SLC5A1,SLC5A11,SLC5A4,SLC7A11,SLC8A2,SLC9A7,SLCO1A2,SLCO2B1,SLIT1,SLIT3,SLITRK5,SMAD9,SMARCA5,SMC3,SMOC2,SMS,SMURF1,SMYD3,SNCAIP,SNX19,SNX4,SOCS2,SORL1,SOX3,SPEG,SPNS3,SPOCK1,SPOCK3,SPON1,SPP2,SPRY1,SPTBN2,SRPX,SRSF3,SSFA2,SSR3,SSTR5,ST18,ST3GAL6,ST6GAL1,ST6GALNAC2,ST6GALNAC5,ST8SIA2,STEAP1B,STK39,STK4,STMN1,STOX2,STX12,SUB1,SV2C,SYN3,SYNJ2,SYT1,SYT5,SYT8,T,TACR3,TAS2R16,TBC1D8,TBPL1,TBX5,TCF4,TERF2IP,TEX9,TFF1,TFG,TG,TGFA,TGFBR1,THBS2,THBS4,THRB,TIMP3,TMEM176B,TMEM187,TMEM237,TMEM35,TMEM40,TMEM42,TMEM50A,TMOD1,TMOD2,TMPO,TMTC2,TNFRSF1B,TNFSF8,TNNI3K,TP53INP2,TP73,TRAF2,TRAF3,TRAM2,TRH,TRIB2,TRIM23,TRIM29,TRIM38,TRPS1,TSPAN13,TSPAN32,TSPAN5,TSPAN7,TSPYL4,TUB,TUBA3C,TUBB2A,TUBB6,TUSC3,TWISTNB,TXNDC12,TYRO3,UBE2E2,UNC5C,USH1C,USP12,USP2,USP38,USP5,VAT1L,VCAM1,VCAN,VIM,VPS8,VSNL1,WAPAL,WDFY2,WDR33,WDR43,WLS,WWOX,ZBBX,ZBTB16,ZBTB20,ZBTB44,ZDHHC6,ZDHHC9,ZEB1,ZFPM2,ZFY,ZIC1,ZIC4,ZNF215,ZNF268,ZNF343,ZNF345,ZNF365,ZNF391,ZNF423,ZNF516,ZNF518B,ZNF688,ZNF696,ZNF721,ZNF800,ZPBP

***Table 2.*** *List of differentially methylated genes in iAs-rev cells that have an oncogenic signature (1059 genes)*

A4GALT,A4GNT,AAGAB,AAK1,ABAT,ABCA2,ABCA5,ABCB4,ABHD5,ACACA,ACADS,ACAP2,ACAT2,ACCN1,ACOX3,ACOXL,ACSM3,ADAM10,ADAM19,ADAMTS12,ADAMTS9,ADAMTSL4,ADCK4,ADCY2,ADCYAP1R1,ADM2,AFF3,AGAP11,AGAP2,AGPAT4,AGTR1,AGXT,AHNAK,AJAP1,AK4,AKT2,ALAD,ALCAM,ALDH1A2,ALDH7A1,ALG13,ALG3,ALK,ALOXE3,ALPK2,ALPL,AMD1,ANGPT4,ANK1,ANK2,ANK3,ANKH,ANKRD11,ANKRD32,ANKRD6,ANLN,APAF1,APBA1,APLNR,APOA1,APOB,APOBEC3A,AQR,ARCN1,ARHGAP25,ARID5B,ARL15,ARMCX1,ARMCX3,ARMCX5,ARNTL,ARPP21,ARRB2,ARRDC2,ARRDC4,ARSJ,ASB3,ASPM,ASTN2,ATF3,ATOH7,ATP13A4,ATP5A1,ATP6V1A,ATP8A1,ATP8A2,ATP8B4,ATRNL1,ATXN2,ATXN7,AUTS2,B2M,B3GALT4,B4GALNT1,BAG3,BAG5,BAI1,BARX2,BBS12,BCAR3,BCAT1,BCL11B,BCL2,BEX2,BIK,BMP7,BMP8B,BNIP2,BPI,BPNT1,BRCA1,BSN,BTBD2,BTBD7,BTD,BTG3,BTN3A2,BZW1,C11orf61,C14orf159,C14orf37,C15orf29,C17orf49,C17orf80,C1orf228,C1QC,C20orf194,C3orf37,C3orf52,C3orf70,C6orf108,C6orf211,C6orf226,C7orf10,C7orf23,CAB39,CACNA1A,CACNA1C,CACNA1I,CACNB3,CACNB4,CADPS,CALCR,CALN1,CAMK2N2,CAMK4,CARS2,CASP2,CAV2,CBFA2T3,CBFB,CBLB,CCDC102B,CCDC26,CCDC88C,CCKBR,CCND1,CCND3,CCNG1,CCNT1,CCNT2,CCPG1,CCR6,CD247,CD320,CD5,CD6,CD83,CD8A,CDC20B,CDC27,CDC6,CDC7,CDCA3,CDH11,CDH12,CDH13,CDH17,CDH19,CDH20,CDH4,CDK6,CDKAL1,CDKL1,CDKN1C,CEACAM21,CEACAM3,CEACAM5,CELF4,CELF6,CEP350,CEP57,CGA,CHD2,CHD7,CHD9,CHFR,CHL1,CHRNA2,CHRNA7,CHST1,CHST10,CHST8,CIB2,CLDN8,CLK1,CLMN,CLN6,CLVS1,CNTN4,CNTNAP5,COL20A1,COL4A3BP,COPS4,COQ10B,COTL1,COX10,CPA2,CPEB2,CPNE4,CPVL,CRCT1,CREB5,CRELD1,CREM,CRISP2,CRLF1,CRMP1,CROT,CRYM,CSGALNACT2,CTBP2,CTNNA2,CTNNA3,CUBN,CUX2,CXCL12,CXCR2,CYB5B,CYFIP2,CYP4F3,D4S234E,DBNDD2,DCAF8,DCC,DCT,DDX39A,DDX60,DGKI,DHRS1,DHTKD1,DHX34,DHX57,DIP2C,DIS3,DKK1,DLGAP2,DLX5,DMRT1,DNAJA1,DNAJC17,DNMBP,DOM3Z,DOPEY2,DPYS,DPYSL2,DPYSL3,DPYSL5,DRD3,DSCAM,DSP,DTX4,DUSP4,DYRK4,EBF1,ECE2,EDARADD,EEPD1,EFCAB6,EFEMP1,EFHD1,EFNA5,EFNB2,EGR4,EHBP1,EHD3,EHMT2,EID1,EIF2AK2,EIF2AK3,EIF2B3,EIF4E,ELMO1,EPB41L3,EPHB2,EPHB3,EPS8L1,ERLIN2,ESRP1,ESRRB,ETNK2,ETS1,EXD3,EXOSC7,EYA4,F2RL1,FA2H,FABP3,FAHD2A,FAM107B,FAM123C,FAM131A,FAM189B,FAM198B,FAM20C,FAM3C,FAM76A,FANCA,FAR2,FAT2,FAT3,FBXL6,FDFT1,FDPS,FGF12,FGF3,FGF9,FHL2,FHOD3,FLI1,FLJ13197,FLNB,FLT4,FMN1,FNDC1,FNDC3B,FOXJ1,FOXO4,FOXP1,FRAS1,FRMD4A,FRMD5,FSHB,FSTL4,FUT3,FZD5,FZD6,GAB1,GAB3,GABRA5,GABRG1,GABRG2,GALC,GALNTL4,GATA3,GATA4,GBA3,GCH1,GDF15,GEMIN5,GGTA1P,GIN1,GINS1,GLUL,GMEB1,GNA14,GNL2,GOLGA2,GPATCH1,GPM6A,GPR12,GPR144,GPR17,GPR173,GPR31,GPR63,GPR68,GRAMD3,GRB14,GRB7,GREB1L,GRIA1,GRID1,GRIN2A,GRIN2B,GRIP1,GRM1,GRP,GRTP1,GSDMC,GTF2H5,GUCY1A3,GULP1,HAUS2,HCRTR2,HDAC4,HDGFRP3,HECW1,HECW2,HELLS,HEPH,HEY2,HIF1A,HIF3A,HINFP,HIST1H2AG,HIST1H2BJ,HIST1H4B,HIST1H4E,HIVEP2,HKDC1,HLADRB6,HMBOX1,HMBS,HMGB2,HMOX1,HMP19,HOMER1,HPCAL1,HPDL,HPN,HRH1,HS6ST1,HSD17B2,HSF2BP,HSP90AA1,HSPA1B,HSPA5,HSPB7,HSPB8,HTR1A,HTR7,HYDIN,ID2,IER5L,IFRD1,IGF2,IGFBP3,IL18R1,IL6R,INPP5A,INPP5B,INTS9,IQCA1,IQCG,IRAK1BP1,IRAK4,IRF2,IRX5,ITGA5,ITIH5,ITPKA,ITPKC,JAM3,JUB,KALRN,KATNB1,KAZN,KCNC2,KCNH2,KCNIP1,KCNIP4,KCNJ10,KCNJ2,KCNJ3,KCNK10,KCNMA1,KCNMB1,KCNN1,KCNN2,KCNS3,KCNV1,KDELR3,KDR,KIAA0895,KIAA0922,KIAA0930,KIAA1009,KIAA1310,KIAA1524,KIAA1967,KIF15,KIF1A,KIF25,KIF2A,KIRREL3,KLF10,KLF3,KLF9,KLHL35,KLK11,KLK12,KLK14,KLK6,KLK7,KNG1,KPNA3,KPNA5,LAMA2,LAMA4,LAMB1,LAPTM4A,LARGE,LCMT1,LCORL,LDB2,LDOC1,LEF1,LHX6,LIMD1,LIN9,LINC00339,LMAN2,LOC157627,LPIN1,LPIN3,LRIG2,LRRC57,LRRC61,LRRFIP1,LSM12,LSR,LTBP3,LY6K,MAD2L1BP,MAGI2,MAGOH,MALL,MAP1B,MAP2K6,MAPK3,MAPRE2,MAPT,MATK,MATN4,MATR3,MBL1P,MCF2L,MCM10,MCTP1,ME3,MED17,MEGF6,METAP1,METTL1,METTL20,METTL3,MIIP,MKI67,MMP15,MNS1,MON1A,MORN2,MPPED1,MPPED2,MRPL13,MRPL30,MRPS18A,MS4A4A,MSL2,MSMO1,MSR1,MTBP,MTHFD1,MTHFR,MTMR10,MTO1,MTPAP,MYBL1,MYF6,MYH14,MYL5,MYO1F,MYOF,MYOM2,MYRIP,MYT1L,NAGA,NAGK,NAV2,NCEH1,NCK2,NDFIP1,NDOR1,NDUFAF4,NDUFV1,NEDD4,NEDD9,NEIL1,NEIL3,NEK1,NELL1,NELL2,NEUROD4,NFIB,NFIX,NFKB2,NFYA,NID1,NKX32,NLRP3,NMBR,NMU,NMUR1,NOL12,NOL4,NOP16,NOS1,NOX3,NPAS2,NPBWR2,NPC2,NPHS2,NPM1,NPPB,NPY5R,NR1H4,NR2E1,NR2F2,NR3C2,NRG1,NRN1,NRP2,NRXN1,NRXN3,NSMAF,NT5DC2,NT5E,NTM,NTN1,NUAK1,NUDCD1,NUDT22,NUMB,NUTF2,NYX,OCA2,ODZ4,OPCML,OR56B1,OSBP2,OSBPL3,OTUD6B,OTUD7A,OTX2,P2RY1,P2RY6,PACRG,PAK1,PAK3,PALLD,PANX1,PAPOLA,PAQR5,PAQR7,PARK2,PARM1,PART1,PAX5,PBX1,PCBD2,PCCB,PCDH8,PCDHA6,PCDHA9,PCDHB12,PCDHB4,PCK2,PCP4,PCSK2,PDE11A,PDE4D,PDE4DIP,PDGFRA,PDLIM3,PDLIM5,PDZD2,PDZRN3,PDZRN4,PEG3,PENK,PEX11B,PEX7,PFKFB1,PFN2,PGK2,PHACTR3,PHKG2,PIAS3,PID1,PIEZO1,PIK3CG,PIK3R1,PILRB,PITPNC1,PKD1,PKIA,PKN1,PKP2,PLBD1,PLCH1,PLOD2,PLS3,PLXNA2,PMPCA,PNLIP,PNPLA3,PNRC1,PODXL,POR,POT1,PPCS,PPFIA3,PPP1R17,PPP1R21,PPP1R9A,PPP2R5B,PPP3CA,PPP3R1,PRAP1,PRB3,PRDM8,PRICKLE1,PRKAB1,PRKACB,PRKAG2,PRKAR2B,PRKCA,PRKCDBP,PRKCE,PRKCH,PRKCQ,PRKG1,PRMT1,PRMT3,PRMT7,PRR3,PRR5L,PRRC2A,PSMD14,PSME4,PSTPIP2,PTGR1,PTPN1,PTPN12,PTPRC,PTPRD,PTPRE,PTPRG,PTPRN2,PTPRS,PTPRZ1,PURG,PUS7L,PVRL3,PXK,PXMP2,PYHIN1,RAB17,RAB6B,RAD18,RAD51AP1,RAD9A,RAI14,RARA,RARB,RASGRP1,RASGRP3,RASSF1,RASSF4,RBFOX1,RCAN2,RCBTB2,RCHY1,RDM1,RECQL,RERE,RERG,RFPL1-AS1,RG9MTD1,RGL1,RGMB,RGS17,RGS4,RGS6,RIMS2,RMI1,RMND1,RMST,RNASEH2B,RNF128,RNF13,RNF144A,RNF39,RNF41,ROBO1,ROCK1,RPL18A,RPL7,RPP25,RPRM,RPS6KA2,RPUSD4,RRAGD,RTN1,RUNX1,RUNX1T1,RUSC2,S100A12,S100A4,S100P,SACM1L,SALL3,SAMD4A,SARDH,SATB2,SBK1,SBNO2,SCAF11,SCAMP1,SCGN,SCN1B,SCN8A,SDCBP,SDCCAG3,SDK2,SEC14L2,SEMA3E,SEMA6D,SEPT11,SERAC1,SERPINA1,SESN1,SF1,SFRP1,SFRP2,SGMS1,SH2D5,SH3GL3,SHANK1,SHQ1,SHROOM3,SIGLEC5,SIRT2,SIX1,SIX4,SLBP,SLC19A2,SLC1A3,SLC1A6,SLC24A3,SLC27A1,SLC27A2,SLC27A5,SLC2A2,SLC2A9,SLC31A1,SLC35D1,SLC38A6,SLC39A4,SLC39A6,SLC48A1,SLC4A4,SLC5A1,SLC5A5,SLC6A15,SLC6A5,SLC7A11,SLC7A3,SLC7A5,SLC8A3,SLC9B2,SLIT1,SLIT2,SLITRK5,SMARCA2,SMARCA5,SMARCD1,SMARCD3,SMPD1,SNORA68,SNRPB2,SNX4,SN8,SOCS2,SOCS5,SOHLH2,SORL1,SOX2OT,SPAG5,SPARCL1,SPRY1,SPTBN4,SQRDL,SRI,SRSF11,SRSF3,SSFA2,SSR3,ST3GAL4,ST6GALNAC5,ST8SIA2,STAC,STAM2,STARD4,STAT3,STAT6,STATH,STEAP1,STK39,STK4,STOML2,STUB1,STXBP2,SUOX,SUV420H1,SVIL,SYDE2,SYK,SYNDIG1,SYNE2,SYNJ2,SYPL1,SYT1,SYT17,TAAR5,TACR1,TAF1D,TAPBP,TBC1D15,TBC1D19,TBC1D2,TBCC,TBPL1,TBX5,TBXAS1,TCEAL2,TCEB1,TCEB3,TCF25,TCF4,TCTN1,TERF2IP,TEX14,TEX2,TFAP2B,TFG,TG,THAP9,THBS2,THRAP3,THRB,TIGD6,TLR10,TLR3,TLR5,TLX3,TMED5,TMEM14A,TMEM159,TMEM173,TMEM174,TMEM176B,TMEM187,TMEM237,TMEM48,TMOD2,TNFAIP3,TNFSF11,TNIK,TNIP3,TNKS,TNNT3,TNPO2,TNR,TOX3,TP53I11,TP53INP2,TP53TG1,TPPP3,TREX1,TRIM23,TRIM46,TRPA1,TRPC1,TSPAN5,TSPAN7,TTC14,TUBGCP2,TXNRD1,UAP1L1,UBE2C,UBE2CBP,UBE2T,UFD1L,UGP2,UGT2A1,UGT8,UNC5C,UNC79,UNKL,USH2A,USP12,USP16,USP38,USP46,USP5,UST,UTP14A,UTRN,VIM,VPS37B,VPS4B,VPS8,VWA3B,WAPAL,WASF3,WDR20,WDR25,WDR43,WDR91,WIPF1,WLS,WNT16,WRN,WT1AS,WWOX,XK,XPO1,XYLB,YIPF2,YWHAE,ZBBX,ZBED3,ZC3H14,ZFPM2,ZHX2,ZMAT3,ZMYND11,ZNF134,ZNF155,ZNF187,ZNF257,ZNF323,ZNF35,ZNF365,ZNF415,ZNF420,ZNF423,ZNF436,ZNF44,ZNF467,ZNF518B,ZNF557,ZNF696,ZNF721,ZNF780B,ZSCAN18,ZW10

***Table 3.*** *List of differentially methylated genes in iAs-rev cells compared to iAsT cells that have an oncogenic signature (893 genes)*

A4GALT,AAK1,ABAT,ABCA2,ABCB4,ABCC4,ABHD5,ABLIM2,ABP1,ACAP2,ACAT2,ACCN1,ACHE,ACOX3,ACOXL,ACSM3,ACTL6B,ADAM11,ADAM17,ADAM22,ADAM3A,ADAMTS12,ADAMTS2,ADAMTSL4,ADAR,ADCK4,ADCY2,ADCYAP1R1,ADD2,AFF3,AGAP2,AGL,AGPAT4,AHNAK,AHNAK2,AHR,AJAP1,AKAP9,AKT3,ALDH1A2,ALOX5,ALPL,AMPH,ANGPT4,ANK1,ANK2,ANKRD11,ANKRD6,ANKS1B,ANO1,ANO2,APBA1,APBA2,APBB2,APOB,APP,AQP9,ARAP3,ARCN1,ARHGEF10L,ARID3B,ARID5B,ARL4A,ARL5A,ARMC9,ARMCX5,ARNTL,ARPP21,ASPM,ASTN2,ATF1,ATP10B,ATP12A,ATP1A3,ATP4A,ATP5A1,ATP5G2,ATP6AP2,ATP6V1A,ATP7B,ATP8A1,ATP8A2,ATP8B2,ATP8B4,ATXN8OS,AUTS2,AXIN1,B3GALT4,BARX2,BCAS1,BCL11B,BIK,BMP3,BMP6,BMP7,BMP8B,BPI,BRCC3,BSN,BTBD11,BTBD2,BTBD7,BTN3A2,BUB1,C10orf128,C11orf61,C14orf37,C15orf2,C15orf29,C15orf60,C1orf21,C1QC,C20orf194,C21orf33,C3orf70,C7orf23,CACNA1C,CACNA1H,CACNB3,CACNB4,CADPS2,CALB2,CALCB,CALCOCO1,CALD1,CALY,CAMK2A,CAND2,CBLB,CCKBR,CCR6,CD24,CD320,CD44,CD6,CD84,CD8A,CDADC1,CDC14A,CDC25B,CDC27,CDH12,CDH13,CDH20,CDH4,CDHR1,CDK6,CDKL1,CDKN1C,CDKN2C,CEACAM21,CEACAM3,CEBPZ,CELF2,CELF4,CELF6,CENPQ,CEP41,CFH,CGNL1,CHGA,CHL1,CHRDL1,CHRNA2,CHRNA7,CHST8,CIB2,CLMN,CLVS1,CMAHP,CMPK2,CNTN5,CNTNAP2,COCH,COL13A1,COL19A1,COL4A3BP,COL6A2,COL6A3,COL9A1,CPA2,CPB2,CPEB1,CPPED1,CPT1A,CRCT1,CREB1,CREM,CRISP2,CRYBB1,CSGALNACT2,CTDSPL,CTNNA2,CTNNA3,CTNND2,CUL4A,CUX2,CWC22,CYFIP2,CYP2B6,CYP39A1,CYP4B1,CYP4F3,CYTIP,DAAM1,DAAM2,DBF4,DCAF8,DCC,DCLK1,DCT,DDX3Y,DDX60L,DEF6,DGKG,DGKI,DIMT1,DIP2C,DISC1,DLG2,DLGAP2,DLX5,DLX6,DNAH3,DNAJC17,DOCK8,DPAGT1,DPYSL2,DPYSL3,DPYSL5,DSCAM,DTX4,DUOX1,DUOX2,DYNC1I1,E2F5,ECE1,EFCAB6,EFEMP1,EFHD1,EGFR,EGR1,EHBP1L1,EHD3,EHMT2,EIF2AK2,EIF4E,ELK3,ELL2,ELMO1,ELOVL6,EMID1,EMR3,ENC1,EOMES,EPHA4,EPHB1,EPHB2,EPHX2,EPS8L1,ERLIN2,ESRRB,ESRRG,ETS1,EYA4,F11,FABP3,FAM107B,FAM118B,FAM123C,FAM131A,FAM131C,FAM167B,FAM208B,FAM20C,FAM3C,FANCA,FASTKD2,FAT2,FAT3,FBLN5,FBN1,FBXO17,FBXO42,FCN3,FDFT1,FEV,FGF12,FGF14,FHOD3,FLI1,FLT4,FNDC3B,FOXM1,FOXP1,FRAS1,FRMD1,FRMD4A,FRZB,FSHR,FSTL4,FUT3,FXYD4,FXYD6,FZD5,GABRA2,GABRA5,GABRB1,GABRG2,GAL,GALNT10,GALNT7,GALNT8,GALNTL4,GAP43,GAPDHS,GATA3,GATA4,GDAP1L1,GFRA1,GGTA1P,GINS1,GLYCAM1,GMEB1,GNA14,GNG7,GNL3L,GOLGA4,GP5,GP6,GPA33,GPC3,GPR12,GPR137B,GPR160,GPR37,GPRC5B,GRAMD3,GRID2,GRIN2A,GRIN2B,GRIP1,GRK4,GRM8,GRPR,GRTP1,GSDMC,GSPT2,GUCY1B2,HARS2,HAS1,HCRTR2,HDAC11,HDAC4,HERC5,HEY2,HIPK2,HIST1H2AG,HIST1H2AK,HIST1H2BN,HMBOX1,HMGB2,HMGN2,HMP19,HNF4G,HOXA5,HOXC11,HPDL,HPN,HS3ST3B1,HS6ST1,HSD17B2,HSD3B7,HSPB7,HTATIP2,HTR1A,HTR1E,HTR2A,HYAL1,IFRD1,IGDCC4,IGF2AS,IGF2BP3,IGSF9B,IKZF1,IL4R,IMPA1,INO80D,IQCA1,IRAK1BP1,IRF2,IRX5,ISG20L2,ISYNA1,ITCH,ITGA9,ITGAE,ITIH5,ITPK1,ITPKC,JAM2,JMJD5,JUB,KALRN,KAT5,KATNB1,KAZN,KCNA2,KCNB2,KCNIP1,KCNIP4,KCNJ1,KCNJ3,KCNK10,KCNQ1,KCNQ4,KCNS3,KCNV1,KDR,KIAA0513,KIAA0930,KIAA1009,KIAA1217,KIAA1310,KIF15,KIF5C,KLF9,KLHL1,KLHL21,KLHL35,KLK10,KLK6,KLK8,KLKB1,KTI12,LAMA2,LAMA3,LARP4,LEF1,LHX6,LILRA4,LIMCH1,LIN28A,LIN9,LMAN2,LMBR1,LMCD1,LMO7,LMOD1,LOC344887,LOR,LOXL1,LPIN1,LRRC20,LSM14A,LSM6,LSP1,LSR,LTBP3,LTF,LYPD1,LYRM5,MAEA,MAGEL2,MAGI2,MAML3,MANEAL,MAP1B,MAP3K1,MAPK10,MAPK11,MAPK3,MAPT,MARS,MAST4,MATK,MCAT,MCF2L,MCTP1,ME3,MECOM,MEF2C,MGLL,MOBP,MOCS1,MPPED1,MRC2,MRPS18A,MS4A12,MS4A3,MSH5,MSL2,MSMO1,MSR1,MST1R,MTHFD2,MTSS1,MTUS1,MYBPC2,MYH14,MYH3,MYH7,MYL5,MYO10,MYO1D,MYO5B,MYOM2,MYRIP,MYT1L,NAPSA,NBEA,NCK2,NDRG1,NDRG2,NDUFC1,NEDD9,NEIL1,NEK1,NELL1,NELL2,NENF,NEUROG3,NFATC2,NFIC,NFKB2,NIPAL3,NKX21,NLRP1,NLRP2,NMBR,NOL4,NOS1,NPAS2,NPC2,NPHS1,NPHS2,NPTX1,NPTXR,NPY1R,NR2E1,NRG1,NRN1,NRXN1,NRXN3,NSMAF,NT5DC2,NTF3,NTM,NTN1,NTNG2,NUP155,NXPH3,OAF,OCA2,ODZ4,ONECUT1,OPCML,OR2H1,ORC6,OSBP2,OSBPL3,OTUB2,OTUD7A,OTX2,PACRG,PAIP2B,PAK3,PAPOLA,PAQR5,PARK2,PARM1,PARP12,PAX1,PAX5,PCDH1,PCDH8,PCDH9,PCDHA9,PCDHB3,PCK2,PCLO,PCOLCE,PDE11A,PDE1B,PDE4C,PDE4D,PDGFB,PDGFRA,PDHA2,PDK1,PDK2,PDLIM3,PDPN,PDYN,PDZD2,PDZRN3,PEG3,PELI2,PHACTR3,PHB,PHF20,PHLDA1,PIK3CD,PINK1,PKD1,PKIB,PKN1,PKP2,PLA2G2A,PLA2G4C,PLEKHA1,PLEKHF1,PLOD2,PLP1,POLR2C,POR,PPIL4,PPM1H,PPP1R15A,PPP1R17,PPP1R9A,PRDM8,PRICKLE1,PRKACB,PRKCE,PRKCH,PRKCQ,PRKG1,PRMT10,PRMT8,PROM1,PRR5L,PRRC2A,PRRT2,PSD4,PTEN,PTGR1,PTK7,PTPRD,PTPRE,PTPRG,PTPRM,PTPRN2,PTPRR,PTPRS,PVRL3,PXK,PYHIN1,RAB21,RAB27A,RAD54L,RAD9A,RAI14,RALGDS,RAPGEF5,RASA3,RASGEF1A,RASGRP1,RASSF1,RASSF4,RBFOX1,RBP7,RCAN1,RCBTB2,RERE,RERG,RFNG,RGS6,RIBC2,RIN2,RMND1,RNASE1,RNASE6,RND3,RNF11,RNF13,RNF144A,RNF41,ROBO1,RPIA,RPS6KA2,RSPH1,RSPO1,RTN1,RXRG,RYR3,S100A16,SALL1,SAMD11,SARDH,SARM1,SARNP,SART3,SATB2,SBNO2,SCAMP1,SCN8A,SDCBP,SDK2,SEC14L2,SEMA3E,SEMA6D,SEPT9,SERHL2,SERPINA1,SERPINA12,SERPINA3,SERPINA5,SERPINA6,SERPINI2,SETMAR,SEZ6L,SF1,SFRP1,SGCG,SGPP2,SHANK1,SHQ1,SIM2,SIX1,SIX4,SLBP,SLC12A5,SLC19A3,SLC1A3,SLC22A17,SLC22A6,SLC24A3,SLC24A5,SLC27A2,SLC2A9,SLC30A1,SLC30A4,SLC35F1,SLC39A4,SLC48A1,SLC5A1,SLC5A4,SLC6A1,SLC7A10,SLC7A5,SLC8A2,SLC8A3,SLCO1A2,SLIT2,SLIT3,SMAP1,SMOC2,SMURF1,SNCAIP,SNN,SNX19,SNX29,SNX4,SOCS2,SORL1,SOX2OT,SOX30,SP140,SP6,SPAG5,SPNS3,SPOCK1,SPON1,SPTBN2,SQRDL,SRPX,SSTR5,ST18,ST3GAL6,ST6GAL1,ST6GALNAC5,ST8SIA1,ST8SIA2,STARD4,STAT3,STATH,STEAP1,STXBP2,SULT4A1,SUMF1,SUSD4,SVIL,SYDE2,SYN3,SYNDIG1,SYNE2,SYPL2,SYT1,TACR3,TBC1D8,TBX5,TCEAL2,TCF4,TEX2,TFAP2B,TG,THAP9,THBS2,THBS4,TIAM1,TIAM2,TIMP3,TLR10,TLR3,TLX3,TM6SF1,TMEM121,TMEM173,TMEM174,TMEM19,TMEM35,TMEM50A,TMEM53,TMOD1,TMOD2,TMPO,TNFRSF19,TNFRSF1B,TNFRSF8,TNFSF8,TNIK,TNNI3K,TNNT3,TNR,TNXB,TP53I11,TP73,TP73AS1,TRAF2,TRAF3,TRAK2,TREM1,TREX1,TRH,TRPA1,TRPS1,TSPAN13,TSPAN32,TSPAN5,TSPYL4,TTYH1,TXNRD1,UBD,UBL3,UGP2,UGT2A1,UNC5C,UNC79,UNKL,USH2A,UST,UTRN,VAMP2,VAT1L,VAV3,VPS37B,VRK1,VSNL1,VWF,WASF3,WDR25,WLS,WNT5A,WNT7A,WWOX,XCL2,YPEL1,YTHDF2,ZBBX,ZBTB16,ZDHHC6,ZDHHC9,ZEB1,ZFP36L1,ZFPM2,ZFY,ZIC1,ZMAT3,ZNF215,ZNF268,ZNF274,ZNF331,ZNF35,ZNF365,ZNF423,ZNF428,ZNF516,ZNF518B,ZNF600,ZNF696,ZNF862,ZSCAN18
